# Design, Synthesis, and Bioactivities of Novel Trifluoromethyl Pyrimidine Derivatives Bearing an Amide Moiety

**DOI:** 10.3389/fchem.2022.952679

**Published:** 2022-07-15

**Authors:** Wenjun Lan, Xuemei Tang, Jia Yu, Qiang Fei, Wenneng Wu, Pei Li, Heng Luo

**Affiliations:** ^1^ Food and Pharmaceutical Engineering Institute, Guiyang University, Guiyang, China; ^2^ State Key Laboratory of Functions and Applications of Medicinal Plants, Guizhou Medical University, Guiyang, China; ^3^ Qiandongnan Engineering and Technology Research Center for Comprehensive Utilization of National Medicine, Kaili University, Kaili, China

**Keywords:** amide, trifluoromethyl pyrimidine, design, synthesis, bioactivity

## Abstract

Twenty-three novel trifluoromethyl pyrimidine derivatives containing an amide moiety were designed and synthesized through four-step reactions and evaluated for their antifungal, insecticidal, and anticancer properties. Bioassay results indicated that some of the title compounds exhibited good *in vitro* antifungal activities against *Botryosphaeria dothidea* (*B. dothidea*), *Phompsis* sp., *Botrytis cinereal* (*B. cinerea*), *Colletotrichum gloeosporioides* (*C. gloeosporioides*), *Pyricutaria oryzae* (*P. oryzae*), and *Sclerotinia sclerotiorum* (*S. sclerotiorum*) at 50 μg/ml. Meanwhile, the synthesized compounds showed moderate insecticidal activities against *Mythimna separata* (*M. separata*) and *Spdoptera frugiperda* (*S. frugiperda*) at 500 μg/ml, which were lower than those of chlorantraniliprole. In addition, the synthesized compounds indicated certain anticancer activities against PC3, K562, Hela, and A549 at 5 μg/ml, which were lower than those of doxorubicin. Notably, this work is the first report on the antifungal, insecticidal, and anticancer activities of trifluoromethyl pyrimidine derivatives bearing an amide moiety.

## Introduction

In recent years, in the field of agricultural production, drug resistance and cross-resistance of existing pesticides continue to develop, and the development of efficient and new pesticides is still an urgent task for scientific researchers (Wei et al., 2021). Due to their unique biological structure, nitrogen-containing heterocyclic compounds have the characteristics of high target specificity and good environmental compatibility, which have become a research hotspot in the creation of new pesticides (Mermer et al., 2021). Among them, pyrimidine is an important lead molecule and an active fragment in the design of biologically active molecules, which is widely used in the design of pesticides and pharmaceutical molecules (Abbas et al., 2021), plant growth regulation (Tsygankova et al., 2018), and other biological activities. Many pyrimidine pesticide molecules are on the market, such as azoxystrobin, fluoxastrobin, pyrimethamine sulfonate, pyrimfen, cyclopropenyl, sulfluramid, and sulfonylureas. Especially in the prevention and treatment of plant fungal diseases, pyrimidine fungicides have become one of the hot spots in the research on new pesticides (Borthakur et al., 2020). In the medicine field, pyrimidine has broad-spectrum biological activity including anti-viral, anti-inflammatory, anti-cancer, and anti-HIV activities (Abdel-Aziz et al., 2021; Abu-Zaied et al., 2021; Ding et al., 2022; Li et al., 2022). Therefore, the molecular design, synthesis, and biological activity of pyrimidine derivatives are still one of the hot research topics in pesticide chemistry (Zhang et al., 2020).

Amide, an important negatively charged organic functional group, was widely present in active compounds, which has a broad spectrum of biological activities and is widely used in the field of pesticides and medicine (Maftei et al., 2015; Bhat et al., 2017). According to statistics, 25% of small-molecule drugs currently in the market contain at least one amide bond in their molecular structure (Kumari et al., 2020). At present, the fungicides of amide compounds have been used for decades, and the application of fungicides is the most effective measure to control phytopathogenic fungi. The use of fungicides can restore a lot of losses every year (Simkhada and Thapa, 2021; Wang R.-X. et al., 2021). The commercial varieties that have been developed include fentanyl, fluopicolide, flutolanil penthiopyrad, boscalid, etc. Amide fungicides can effectively prevent and control sheath blight, scab, and sclerotinia on crops such as wheat, corn, rapeseed, and rice. It can also effectively prevent and control fusarium wilt on tomato and potato diseases (AL-Shammri et al., 2022). In our previous work, a series of synthesized pyrimidine-containing substituted amide derivatives exhibited good antifungal activity.

**FIGURE 1:**
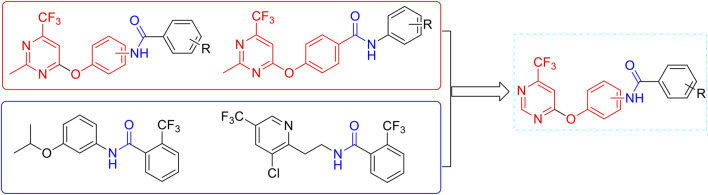
Design of the target molecules.

Based on the aforementioned considerations and our previous works (Wu et al., 2019; Wu et al., 2020; Wu et al., 2021; Yu et al., 2021), the amide is linked to the trifluoromethyl pyrimidine backbone through an oxygen ether. Finally, we designed and synthesized a novel series of trifluoromethyl pyrimidine derivatives containing an amide moiety due to the molecularly active splicing strategy (Figure 1).

## Materials and Methods

### General Information

Melting points (m.p.) of the target compounds were tested on an XT-4 binocular microscope (Beijing Tech Instrument Co., China). ^1^H nuclear magnetic resonance (NMR) and ^13^C nuclear magnetic resonance (NMR) (solvent DMSO-*d*_6_) spectral analyses were performed on a Bruker AvanceNEO spectrometer (600 MHz, Bruker, Germany) at room temperature. High-resolution mass spectrometry (HRMS) data were obtained on a Thermo Scientific Q Exactive Focus instrument (Thermo Fisher Scientific, Unites States). Analytical thin-layer chromatography (TLC) was prepared on silica gel GF_254_.

### Preparation of the Intermediates **2**–**4**

**SCHEME 1:**
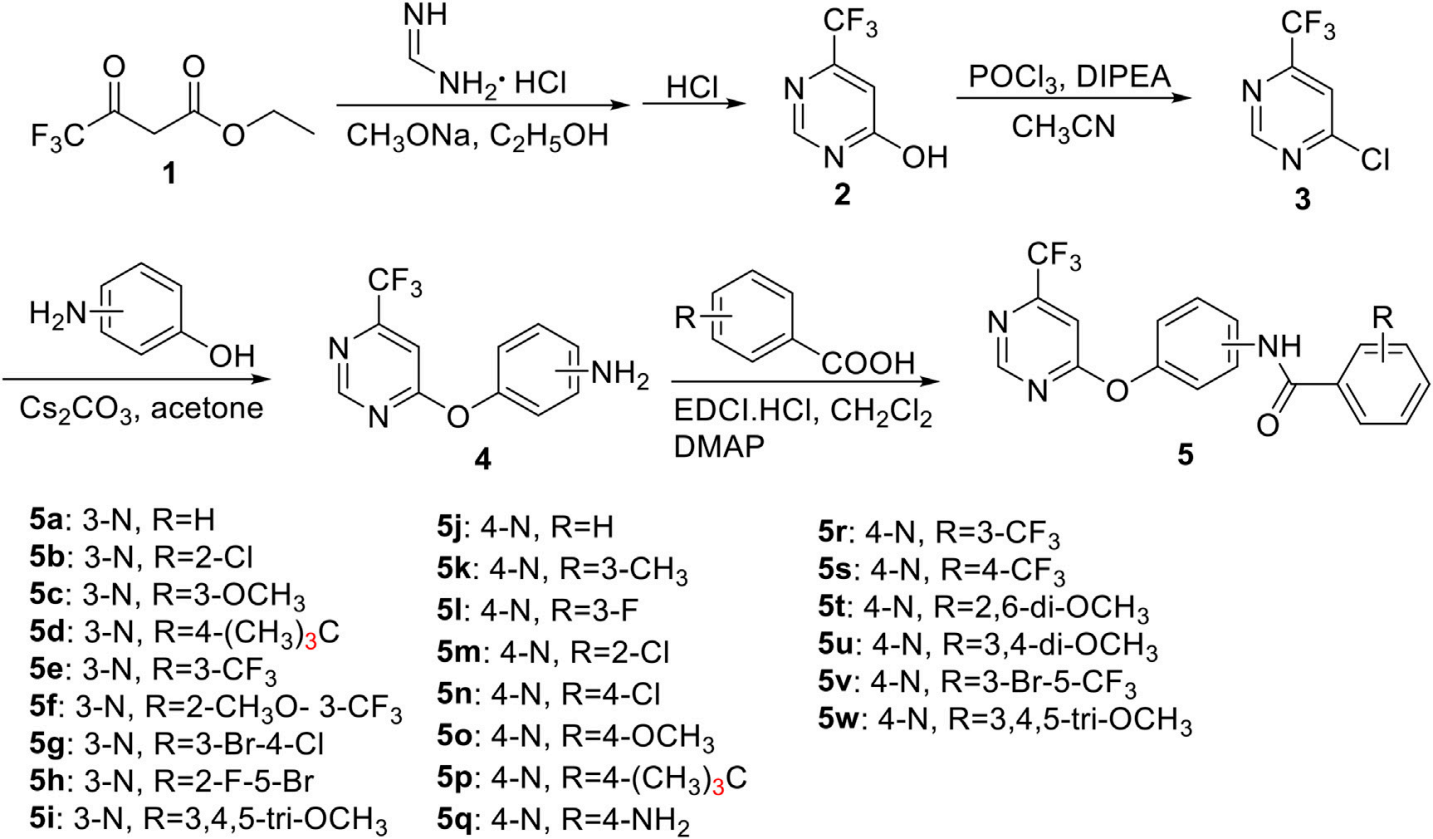
Synthetic routes of the target compounds **5a–5w**.

Intermediates **2** and **3** were prepared using our previous research method in Scheme 1 (Wu et al., 2019).

To a 100-ml three-necked bottle, intermediate **3** (20 mmol), KI (0.2 mmol), Cs_2_CO_3_ (30 mmol), and acetone (50 ml) were stirred under ice bath conditions. Then, dissolved 3-aminophenol or 4-aminophenol (20 mmol) in acetone (10 ml) was added dropwise, which continued to react for 7–8 h at 25°C. The chromatographic column was installed and eluted with petroleum ether and ethyl acetate in different proportions to gain intermediate **4**.

3-((6-(Trifluoromethyl)pyrimidin-4-yl)oxy)aniline (**4a**). White solid; yield 62.5%; m. p. 65–67°C; ^1^H NMR (600 MHz, DMSO-d_6_, ppm) δ: 8.94 (s, 1H, pyrimidine-H), 7.29 (s, 1H, pyrimidine-H), 7.06 (t, 2H, *J* = 7.8 Hz, Ph-H), 6.62 (d, 1H, *J* = 7.8 Hz, Ph-H), 6.42 (t, 1H, *J* = 2.4 Hz, Ph-H), 6.38 (d, 1H, *J* = 7.8 Hz, Ph-H), 5.40 (s, 2H, NH_2_); ^13^C NMR (125 MHz, DMSO-d_6_, ppm)δ: 171.50, 169.88, 156.13 (q,*J* = 35.4 Hz), 147.42, 142.18, 122.26, 121.82 (q, *J* = 273.3 Hz), 114.96, 102.48; HRMS (ESI) calculated for C_11_H_9_ON_3_F_3_ [M + H]^+^: 256.06839, found: 256.06922.

4-((6-(Trifluoromethyl)pyrimidin-4-yl)oxy)aniline (**4b**). White solid; yield 70.6%; m. p. 85–87°C; ^1^H NMR (600 MHz, DMSO-d_6_, ppm)*δ*: 8.96 (s, 1H, pyrimidine-H), 7.55 (s, 1H, pyrimidine-H), 6.93 (d, 2H, *J* = 9.0 Hz, Ph-H), 6.65 (d, 2H, *J* = 8.5 Hz, Ph-H), 5.17 (s, 2H, NH_2_); ^13^C NMR (125 MHz, DMSO-d_6_, ppm) *δ*: 171.41, 159.88, 156.09 (q, *J* = 35.1 Hz), 147.45, 142.06, 122.17, 121.82 (q, *J* = 272.7 Hz), 114.83, 105.83; HRMS (ESI) calculated for C_11_H_9_ON_3_F_3_ [M + H]^+^: 256.06839, found: 256.06910.

### Preparation of the Target Compounds **5a**–**5w**

To a 50-ml three-necked bottle, the key intermediate **4** (0.02 mol), aromatic acid (0.024 mol), and dimethylaminopyridine (DMAP, 0.0004 mol), dissolved in dichloromethane (20 ml), and 1-(3-dimethylaminopropyl)-3-ethylcarbodiimide hydrochloride (EDCI, 0.03 mol) were added. The reactions were stirred at 25°C for 8–10 h. Then, the solvent was evaporated under vacuum and the residue was purified by column chromatography (ethyl acetate/petroleum ether = 10/1) to obtain the target compounds **5a**–**5w**.

*N*-(3-((6-(trifluoromethyl)pyrimidin-4-yl)oxy)phenyl)benzamide (**5a**). White solid; yield 53.4%; m. p. 125–127°C; ^1^H NMR (600 MHz, DMSO-d_6_, ppm) *δ*: 10.46 (s, 1H, -CONH-), 8.99 (s, 1H, pyrimidine), 7.83 (d, 2H, *J* = 7.2 Hz, Ph-H), 7.71 (t, 1H, *J* = 1.8 Hz, Ph-H), 7.78 (s, 1H, Ph-H), 7.72 (d, 1H, *J* = 8.4 Hz, Ph-H), 7.62 (t, 1H, *J* = 7.8 Hz, Ph-H), 7.56 (t, 1H, *J* = 7.8 Hz, Ph-H), 7.49 (t, 1H, *J* = 7.8 Hz, Ph-H), 7.04 (dd, 1H, *J*_1_ = 1.8 Hz, *J*_2_ = 8.4 Hz, Ph-H); ^13^C NMR (150 MHz, DMSO-d_6_, ppm) δ: 170.61, 166.25, 159.84, 156.13 (q, *J* = 35.6 Hz), 152.21, 141.21, 135.17, 132.19, 130.37, 128.88, 128.15, 121.81 (q, *J* = 273.3 Hz), 120.35, 118.30, 117.88, 117.13, 113.70, 106.66, 60.58, 55.79; HRMS (ESI) calculated for C_18_H_12_O_2_N_3_F_3_ [M + Na]^+^: 382.07681, found: 382.07725.

### Crystal Data and Structure Determination

**FIGURE 2:**
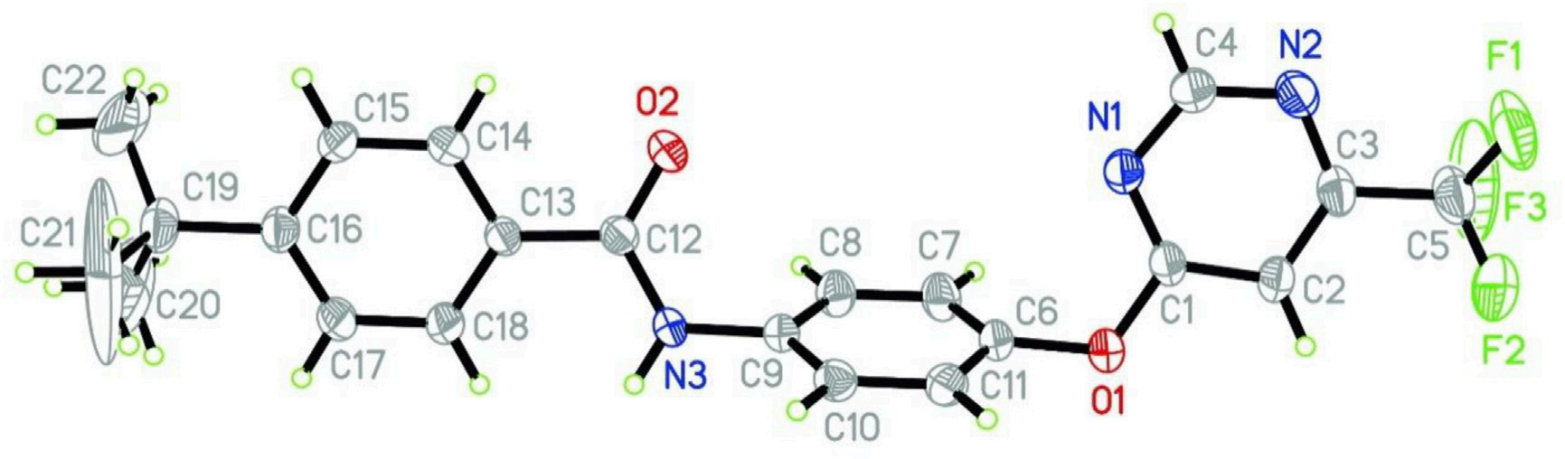
X-ray crystal structure of **5p**.

Single crystals of compound **5q** (deposition CCDC1965888) (DOI: 10.5517/ccdc.csd.cc23znrj) for X-ray diffraction were acquired from absolute ethanol by slow evaporation at 25°C. The crystallographic parameters are displayed in Supplementary Table S1. Supplementary Table S1 showed that 3,107 independent reflections with the range of 2.072°≤ *θ* ≤ 22.729° were obtained. Compound **5q** crystallized in the monoclinic system and the space group is P2 (1)/c. The crystallographic parameters are as follows: *a* = 17.2341 (15) Å, *b* = 12.1803 (11) Å, *c* = 10.5015 (9) Å, *α* = 90°, *β* = 97.021 (2)°, *γ* = 90°, *μ* = 0.106 mm^−1^, *V* = 2187.9 (3) Å^3^, *Z* = 4, *D*_*c*_ = 1.316 g cm^−3^, *F* (000) = 904.0, goodness of fit on *F* ^2^ = 1.000, *R*_1_ = 0.1469, and *wR*_2_ = 0.2676. Meanwhile, the crystal structure of **5q** was shown in Figure 1. Figure 2 showed that the crystal structure of compound **5q** is monoclinic and contains two plane subunits of 6-(trifluoromethyl)pyrimidine and benzamide.

### *In Vitro* Antifungal Activity Test

The antifungal activities against *Botryosphaeria dothidea* (*B. dothidea*), *Phompsis* sp., *Botrytis cinereal* (*B. cinerea*), *Colletotrichum gloeosporioides* (*C. gloeosporioides*), *Pyricutaria oryzae* (*P. oryzae*), and *Sclerotinia sclerotiorum* (*S. sclerotiorum*) of compounds **5a**–**5w** at 50 μg/ml were determined by the typical mycelium growth rate method (Du et al., 2021; Wang W. et al., 2021), and tebuconazole was used as a positive control.

### Insecticidal Activity Test

The insecticidal activities against *Spdoptera frugiperda* (*S. frugiperda*) and *Mythimna separata* (*M. separata*) of compounds **5a**–**5w** at 500 µg/mL were conducted according to the research method in the literature (Wang et al., 2020). Chlorantraniliprole was used as a positive control. Three replicates were performed for each treatment. The corrected mortality rate of compounds **5a**–**5w** were evaluated by the Abbott’s formula.

Corrected mortality rate (%)=(Mortality rate of treatment group-Mortality rate of control group)/(1-Mortality rate of control group)×100

### Anticancer Activity Test

The anticancer activities against the cells of compounds **5a**–**5w** at 5 µg/mL were conducted based on the MTT method (El-Dydamony et al., 2022). Doxorubicin was used as a positive control. Each treatment was repeated 3 times. The corrected mortality rates for compounds **5a**–**5w** were determined using the aforementioned formula.

## Results and Discussion

### Chemistry

Using ethyl trifluoroacetoacetate as the initial reagent, as shown in Scheme 1, a series of novel trifluoromethyl pyrimidine derivatives bearing an amide moiety were designed and synthesized via four-step reactions with the yields of 20.2–60.8% and the target compounds were characterized by ^1^H NMR, ^13^C NMR, X-ray diffraction, and HRMS.

The ^1^H NMR signals for compound **5a**, a singlet appears 10.46 ppm indicates the presence of the -CONH- group. The CH proton of the 6-trifluoromethyl pyrimidine ring is located as two singlets at 8.99 and 7.78 ppm. Meanwhile, in the ^13^C NMR data of compound **5a**, two quartets at 156.13and121.81 ppm indicated the presence of -CF_3_and C-CF_3_ as characteristic peaks in the pyrimidine fragment. In addition, compound **5a** was confirmed correctly with the [M + Na]^+^ peaks by HRMS data.

### Antifungal Activity Test *In Vitro*

Table 1 shows that compounds **5b**, **5j**, and **5l** revealed excellent *in vitro* antifungal activity against *B. cinerea*, with the inhibition rates of 96.76, 96.84, and 100%, respectively, which were equal to or even better than that of tebuconazole (96.45%). Meanwhile, compound **5v** had an inhibitory effect (82.73%) against *S. sclerotiorum* equal to that of tebuconazole (83.34%). Nevertheless, compounds **5a**–**5w** revealed lower *in vitro* antifungal activities against *B. Dothidea* (60.48–90.12%), *Phomopsis* sp. (54.37–82.34%), *C. gloeosporioides* (35.12–69.75%), and *P. oryzae* (30.11–63.91%) than those of tebuconazole.

**TABLE 1:** The antifungal activities of the title compounds **5a**−**5w** at 50 μg/ml.

Compound	Inhibition rate (%)					
	*B. dothidea*	*Phomopsis* sp	*B. cinerea*	*C. gloeosporioides*	*P. oryzae*	*S. sclerotiorum*
**5a**	77.25 ± 3.15	59.22 ± 1.39	92.43 ± 3.25	53.89 ± 1.09	38.92 ± 1.86	72.18 ± 3.02
**5b**	88.72 ± 3.83	54.37 ± 1.67	96.76 ± 3.83	51.88 ± 3.61	43.64 ± 1.99	75.82 ± 2.08
**5c**	76.82 ± 3.22	70.73 ± 2.93	89.88 ± 2.89	64.12 ± 2.26	41.34 ± 2.31	76.28 ± 2.05
**5d**	67.34 ± 1.39	58.17 ± 1.94	71.35 ± 2.45	46.53 ± 1.23	36.05 ± 2.94	72.73 ± 1.12
**5e**	78.60 ± 3.70	75.19 ± 3.55	87.68 ± 3.17	49.11 ± 1.08	63.91 ± 1.34	75.45 ± 1.64
**5f**	73.87 ± 2.48	70.37 ± 3.03	89.04 ± 3.15	69.75 ± 1.53	52.49 ± 2.07	79.21 ± 2.30
**5g**	87.95 ± 4.47	85.70 ± 3.87	88.17 ± 1.61	43.17 ± 2.07	47.82 ± 2.64	74.00 ± 1.22
**5h**	71.81 ± 2.24	62.42 ± 2.71	89.51 ± 3.24	41.98 ± 3.51	61.48 ± 1.69	77.82 ± 1.29
**5i**	77.96 ± 3.42	64.14 ± 1.35	87.97 ± 3.58	43.56 ± 1.64	42.69 ± 2.81	57.27 ± 2.40
**5j**	89.89 ± 1.64	77.48 ± 2.63	96.84 ± 2.01	60.01 ± 1.09	41.65 ± 1.38	76.18 ± 1.05
**5k**	75.62 ± 2.18	65.34 ± 1.87	81.54 ± 1.25	41.98 ± 1.37	51.23 ± 3.05	70.36 ± 2.14
**5l**	88.84 ± 1.76	70.34 ± 2.25	100.00	39.21 ± 2.50	44.02 ± 1.96	65.45 ± 3.24
**5m**	74.26 ± 1.18	64.20 ± 2.35	90.40 ± 2.16	35.12 ± 1.79	30.11 ± 1.61	64.28 ± 2.18
**5n**	82.35 ± 1.15	68.23 ± 1.72	94.63 ± 3.46	41.58 ± 3.01	35.67 ± 3.81	73.27 ± 3.24
**5o**	86.49 ± 1.29	79.70 ± 1.53	92.84 ± 1.83	66.87 ± 1.94	40.16 ± 1.28	79.26 ± 3.58
**5p**	71.61 ± 2.34	58.81 ± 2.65	65.87 ± 1.32	52.15 ± 1.38	31.06 ± 2.54	58.27 ± 1.75
**5q**	60.48 ± 1.15	63.82 ± 2.83	76.48 ± 1.37	52.48 ± 2.00	49.70 ± 1.42	61.82 ± 1.09
**5r**	83.65 ± 1.70	79.69 ± 1.58	90.56 ± 1.17	41.58 ± 3.52	35.67 ± 2.62	73.27 ± 2.48
**5s**	90.12 ± 1.37	81.26 ± 1.19	93.47 ± 2.54	40.20 ± 1.08	37.33 ± 1.34	69.09 ± 2.81
**5t**	81.21 ± 1.20	79.65 ± 3.49	93.67 ± 1.67	50.05 ± 1.69	61.10 ± 2.20	72.64 ± 1.84
**5u**	76.82 ± 3.22	70.73 ± 2.93	89.88 ± 2.89	55.05 ± 2.22	47.25 ± 3.04	60.00 ± 2.19
**5v**	82.80 ± 1.44	82.34 ± 1.06	92.64 ± 1.13	51.88 ± 1.52	60.15 ± 1.11	82.73 ± 1.84
**5w**	75.86 ± 1.23	68.56 ± 2.35	92.31 ± 2.46	50.28 ± 0.98	47.36 ± 1.56	64.13 ± 2.27
Tebuconazole	100.00	100.00	96.45 ± 1.82	100.00	100.00	83.34 ± 1.18

### Insecticidal Activity Test

Table 2 shows that compounds **5a**–**5w** indicated certain insecticidal activities against *S. frugiperda* and *M. separata* at 500 μg/ml, with the mortality rates of 13.3–90.0% and 16.7–86.7%, respectively, which were lower than those of chlorantraniliprole. Especially, compound **5w** revealed fine insecticidal activities against *Spdoptera frugiperda* and *Mythimna separata* with the mortality rates of 90.0% and 86.7%, respectively. Meanwhile, compound **5o** and **5t** demonstrated moderate mortality rates of 80.0% and 83.3% against *Spdoptera frugiperda.*

**TABLE 2:** The insecticidal activities of the title compounds **5a**−**5w** at 500 μg/ml.

Compound	Mortality rate (%)	
	*S. frugiperda*	*M. separata*
5a	30.00 ± 1.60	16.70 ± 1.50
5b	13.33 ± 1.00	36.67 ± 1.80
5c	70.00 ± 1.50	60.00 ± 2.20
5d	50.00 ± 2.00	36.67 ± 2.00
5e	46.67 ± 1.50	30.00 ± 1.30
5f	60.00 ± 2.80	56.67 ± 1.20
5g	46.67 ± 1.00	30.00 ± 1.00
5h	50.00 ± 2.20	20.00 ± 1.50
5i	53.33 ± 1.20	33.33 ± 1.20
5j	26.67 ± 1.40	26.67 ± 1.80
5k	40.00 ± 2.00	50.00 ± 1.00
5l	26.67 ± 1.50	70.00 ± 2.20
5m	30.00 ± 1.00	16.67 ± 1.80
5n	40.00 ± 1.30	20.00 ± 1.20
5o>	80.00 ± 2.10	67.67 ± 2.50
5p	56.67 ± 1.00	46.67 ± 1.60
5q	43.33 ± 1.20	30.00 ± 2.00
5r	50.00 ± 1.80	27.00 ± 1.50
5s	66.67 ± 2.47	33.33 ± 1.30
5t	83.33 ± 1.56	20.00 ± 2.30
5u	73.33 ± 1.31	80.00 ± 1.50
5v	73.33 ± 2.03	43.33 ± 1.35
5w	90.00 ± 1.80	86.67 ± 2.42
Chlorantraniliprole	100	100

### Anticancer Activity Test

Table 3 shows that compounds **5a**–**5w** indicated certain anticancer activities against PC3 (0–64.20%), K562 (0–37.80%), Hela (0–48.25%), and A549 (0–40.78%) at 5 μg/ml which were lower than those of doxorubicin. Particularly, compounds **5l**, **5n**, **5o**, **5r**, and **5v** expressed moderate anticancer activities against PC3 with the inhibition rates of 54.94, 51.71, 50.52, 55.32, and 64.20%, respectively.

**TABLE 3:** The anticancer activities of the title compounds **5a**−**5w** at 5 μg/ml.

Compound	Mortality rate (%)			
	PC3	K562	Hela	A549
**5a**	0	10.03 ± 1.03	3.82 ± 2.64	15.76 ± 1.30
**5b**	15.53 ± 1.08	0	2.42 ± 1.06	13.65 ± 2.43
**5c**	0	19.59 ± 1.30	5.98 ± 1.09	13.74 ± 1.76
**5d**	5.80 ± 2.13	4.73 ± 2.15	11.37 ± 2.15	13.60 ± 2.80
**5e**	0	5.30 ± 1.16	0.55 ± 2.24	9.23 ± 1.32
**5f**	4.08 ± 1.67	21.88 ± 2.24	4.23 ± 1.25	12.47 ± 2.35
**5g**	0	15.52 ± 1.61	2.75 ± 1.60	14.51 ± 2.35
**5h**	0	10.98 ± 1.14	0	0.22 ± 1.05
**5i**	3.98 ± 1.22	3.53 ± 1.95	3.58 ± 1.69	15.15 ± 2.64
**5j**	31.01 ± 1.90	30.99 ± 2.06	25.22 ± 2.37	22.75 ± 2.94
**5k**	34.20 ± 2.01	37.80 ± 1.95	34.50 ± 1.89	28.50 ± 2.22
**5l**	54.94 ± 1.51	15.47 ± 1.38	32.20 ± 2.10	37.35 ± 2.00
**5m**	0	0	0	0
**5n**	51.71 ± 1.20	17.11 ± 1.52	30.78 ± 1.45	40.78 ± 1.09
**5o**	50.52 ± 1.22	19.40 ± 1.64	39.54 ± 2.22	38.78 ± 2.26
**5p**	9.20 ± 1.45	7.30 ± 2.01	20.40 ± 1.70	16.80 ± 3.26
**5q**	46.11 ± 1.54	31.40 ± 3.12	37.90 ± 3.01	42.42 ± 1.33
**5r**	55.32 ± 1.35	15.63 ± 0.96	34.65 ± 1.87	41.36 ± 1.51
**5s**	22.35 ± 1.16	5.28 ± 1.38	25.14 ± 2.03	15.24 ± 1.67
**5t**	0.37 ± 1.23	12.46 ± 2.26	6.87 ± 2.07	8.41 ± 2.83
**5u**	31.01 ± 1.64	30.99 ± 1.34	25.22 ± 2.35	22.75 ± 2.90
**5v**	64.20 ± 1.12	24.00 ± 1.92	48.25 ± 1.17	34.20 ± 2.31
**5w**	36.20 ± 1.36	35.40 ± 1.83	32.20 ± 2.62	31.80 ± 2.08
Doxorubicin	94.68 ± 1.05	72.81 ± 1.54	80.43 ± 1.36	89.57 ± 2.07

The preliminary structure–activity relationship showed that most compounds exhibited good activities against *B. dothidea*, *Phomopsis* sp*.*, and *B. cinerea*. Especially for *B. cinerea*., majority of the compounds revealed inhibition rates higher than 80%. The inhibition rate of compound **5l** was even up to 100%, which was exceeded by the control drug tebuconazole (96.45%). Excellent inhibitory activities also indicated the potential of these compounds as candidates or leading structure against *B. cinerea*.

## Conclusion

In summary, twenty-three novel trifluoromethyl pyrimidine derivatives including an amide moiety were prepared based on amide and pyrimidine pharmacophore, and their structures were confirmed by ^1^H NMR, ^13^C NMR, X-ray diffraction, and HRMS determination. The preliminary biological activity screening indicated that most of the title compounds exhibited moderate to excellent antifungal and insecticidal activities. This study demonstrated the potential of trifluoromethyl pyrimidine derivatives including an amide moiety as the effective antifungal and insecticidal agents for crop protection and should be used as the reference for future research.

## Data Availability

The original contributions presented in the study are included in the article/Supplementary Material; further inquiries can be directed to the corresponding authors.

## References

[B1] Abbas N. Swamy P. M. G. Dhiwar P. Patel S. Giles D. (2021). Development of Fused and Substituted Pyrimidine Derivatives as Potent Anticancer Agents (A Review). Pharm. Chem. J. 54 (12), 1215–1226. 10.1007/s11094-021-02346-8

[B2] Abdel-Aziz S. A. Taher E. S. Lan P. Asaad G. F. Gomaa H. A. M. El-Koussi N. A. (2021). Design, Synthesis, and Biological Evaluation of New Pyrimidine-5-Carbonitrile Derivatives Bearing 1,3-Thiazole Moiety as Novel Anti-Inflammatory EGFR Inhibitors with Cardiac Safety Profile. Bioorg. Chem. 111, 104890. 10.1016/j.bioorg.2021.104890 33872924

[B3] Abu-Zaied M. A. Elgemeie G. H. Mahmoud N. M. (2021). Anti-Covid-19 Drug Analogues: Synthesis of Novel Pyrimidine Thioglycosides as Antiviral Agents against SARS-COV-2 and Avian Influenza H5N1 Viruses. ACS Omega 6 (26), 16890–16904. 10.1021/acsomega.1c01501 34250348 PMC8247785

[B4] AL-Shammri K. N. Elkanzi N. A. A. Arafa W. A. A. Althobaiti I. O. Bakr R. B. Moustafa S. M. N. (2022). Novel Indan-1,3-Dione Derivatives: Design, Green Synthesis, Effect against Tomato Damping-Off Disease Caused by Fusarium Oxysporum and In Silico Molecular Docking Study. Arab. J. Chem. 15 (5), 103731. 10.1016/j.arabjc.2022.103731

[B5] Bhat A. R. Dongre R. S. Naikoo G. A. Hassan I. U. Ara T. (2017). Proficient Synthesis of Bioactive Annulated Pyrimidine Derivatives: A Review. J. Taibah Univ. Sci. 11 (6), 1047–1069. 10.1016/j.jtusci.2017.05.005

[B6] Borthakur S. K. Kalita P. K. Borthakur S. (2020). Synthesis and Antifungal Activities of 3,5-Diphenyl-7-Amino-[1,3]-Thiazolo[3,2-A]pyrimidine-6-Nitrile Derivatives. J. Heterocycl. Chem. 57 (3), 1–5. 10.1002/jhet.3863

[B7] Ding L. Pannecouque C. De Clercq E. Zhuang C. Chen F.-E. (2022). Discovery of Novel Pyridine-Dimethyl-Phenyl-DAPY Hybrids by Molecular Fusing of Methyl-Pyrimidine-DAPYs and Difluoro-Pyridinyl-DAPYs: Improving the Druggability toward High Inhibitory Activity, Solubility, Safety, and PK. J. Med. Chem. 65 (3), 2122–2138. 10.1021/acs.jmedchem.1c01676 35073089

[B8] Du S. Yuan Q. Hu X. Fu W. Xu Q. Wei Z. (2021). Synthesis and Biological Activity of Novel Antifungal Leads: 3,5-Dichlorobenzyl Ester Derivatives. J. Agric. Food Chem. 69 (51), 15521–15529. 10.1021/acs.jafc.1c04022 34928597

[B9] El-Dydamony N. M. Abdelnaby R. M. Abdelhady R. Ali O. Fahmy M. I. R. Fakhr Eldeen R. (2022). Pyrimidine-5-Carbonitrile Based Potential Anticancer Agents as Apoptosis Inducers through PI3K/AKT Axis Inhibition in Leukaemia K562. J. Enzym. Inhib. Med. Chem. 37 (1), 895–911. 10.1080/14756366.2022.2051022 PMC8967206 35345960

[B10] Kumari S. Carmona A. V. Tiwari A. K. Trippier P. C. (2020). Amide Bond Bioisosteres: Strategies, Synthesis, and Successes. J. Med. Chem. 63 (21), 12290–12358. 10.1021/acs.jmedchem.0c00530 32686940 PMC7666045

[B11] Li W. Zhang J. Wang M. Dong R. Zhou X. Zheng X. (2022). Pyrimidine-Fused Dinitrogenous Penta-Heterocycles as a Privileged Scaffold for Anti-Cancer Drug Discovery. Curr. Top. Med. Chem. 22 (4), 284–304. 10.2174/1568026622666220111143949 35021973

[B12] Maftei C. V. Fodor E. Jones P. G. Freytag M. Franz M. H. Kelter G. (2015). N-Heterocyclic Carbenes (NHC) with 1,2,4-Oxadiazole-Substituents Related to Natural Products: Synthesis, Structure and Potential Antitumor Activity of Some Corresponding Gold(I) and Silver(I) Complexes. Eur. J. Med. Chem. 101, 431–441. 10.1016/j.ejmech.2015.06.053 26185007

[B13] Mermer A. Keles T. Sirin Y. (2021). Recent Studies of Nitrogen Containing Heterocyclic Compounds as Novel Antiviral Agents: A Review. Bioorg. Chem. 114, 105076. 10.1016/j.bioorg.2021.105076 34157555

[B14] Simkhada K. Thapa R. (2021). Rice Blast, A Major Threat to the Rice Production and its Various Management Techniques. Turk. JAF Sci.Tech. 10 (2), 147–157. 10.24925/turjaf.v10i2.147-157.4548

[B15] Tsygankova V. Andrusevich Y. Shtompel O. Kopich V. Solomyanny R. Bondarenko O. (2018). Phytohormone-Like Effect of Pyrimidine Derivatives on Regulation of Vegetative Growth of Tomato. Int. J. Bot. Stud. 2 (3), 91–102. Available at: https://www.researchgate.net/publication/324123283 .

[B24] Wang B. Wang H. Liu H. Xiong L. Yang N. Zhang Y. (2020). Synthesis and Structure-Insecticidal Activity Relationship of Novel Phenylpyrazole Carboxylic Acid Derivatives Containing Fluorine Moiety. Chinese Chemical Letters.. 10.1016/j.cclet.2019.07.064

[B16] Wang R.-X. Du S.-S. Wang J.-R. Chu Q.-R. Tang C. Zhang Z.-J. (2021). Design, Synthesis, and Antifungal Evaluation of Luotonin A Derivatives against Phytopathogenic Fungi. J. Agric. Food Chem. 69 (48), 14467–14477. 10.1021/acs.jafc.1c04242 34843231

[B17] Wang W. Cheng X. Cui X. Xia D. Wang Z. Lv X. (2021). Synthesis and Biological Activity of Novel Pyrazolo[3,4‐d]pyrimidin‐4‐one Derivatives as Potent Antifungal Agent. Pest Manag. Sci. 77 (7), 3529–3537. 10.1002/ps.6406 33837653

[B18] Wei L. Zhang J. Tan W. Wang G. Li Q. Dong F. (2021). Antifungal Activity of Double Schiff Bases of Chitosan Derivatives Bearing Active Halogeno-Benzenes. Int. J. Biol. Macromol. 179, 292–298. 10.1016/j.ijbiomac.2021.02.184 33652045

[B19] Wu W. Chen M. Fei Q. Ge Y. Zhu Y. Chen H. (2020). Synthesis and Bioactivities Study of Novel Pyridylpyrazol Amide Derivatives Containing Pyrimidine Motifs. Front. Chem. 8, 522. 10.3389/fchem.2020.00522 32850614 PMC7411148

[B22] Wu W.-N. Chen M.-H. Wang R. Tu H.-T. Yang M.-F. Ouyang G.-P. (2019). Novel Pyrimidine Derivatives Containing an Amide Moiety: Design, Synthesis, and Antifungal Activity. Chem. Pap. 73, 719–729. 10.1007/s11696-018-0583-7

[B23] Wu W.-N. Lan W. J. Wu C.-Y. Fei Q. (2021). Synthesis and Antifungal Activity of Pyrimidine Derivatives Containing an Amide Moiety. Front. Chem. 9, 695628. 10.3389/fchem.2021.695628 34322475 PMC8311460

[B20] Yu X. Lan W. Chen M. Xu S. Luo X. He S. (2021). Synthesis and Antifungal and Insecticidal Activities of Novel *N*-Phenylbenzamide Derivatives Bearing a Trifluoromethylpyrimidine Moiety. J. Chem. 2021, 8370407. 10.1155/2021/8370407

[B21] Zhang N. Huang M.-Z. Liu A.-P. Liu M.-H. Li L.-Z. Zhou C.-G. (2020). Design, Synthesis, and Insecticidal/Acaricidal Evaluation of Novel Pyrimidinamine Derivatives Containing Phenyloxazole Moiety. Chem. Pap. 74, 963–970. 10.1007/s11696-019-00932-5

