# Supplementary material for: Design, Synthesis, and Bioactivities of Novel Trifluoromethyl Pyrimidine Derivatives Bearing an Amide Moiety
Source: Front Chem. 2022 Jul 15;10:952679. doi: 10.3389/fchem.2022.952679 (PMC9334529; doi:10.3389/fchem.2022.952679)
Supplement: Supplementary file 1 [file Presentation1.pdf]

Design, Synthesis, and Bioactivities of Novel Trifluoromethyl Pyrimidine Derivatives bearing an Amide Moiety

Wenjun Lan[1], Xuemei Tang[1], Jia Yu[2], Qiang Fei[1], Wenneng Wu[1,*], Pei Li[3,*], Heng Luo[2,*]

[1]Food and Pharmaceutical Engineering Institute, Guiyang University, Guiyang 550003, PR China
[2]State Key Laboratory of Functions and Applications of Medicinal Plants, Guizhou Medical University, Guiyang 550014, China.
[3]Qian dong nan Engineering and Technology Research Center for Comprehensive Utilization of National Medicine, Kaili University, Kaili 556011, PR China
Wenjun Lan and Xuemei Tang contributed equally to this article
*** Correspondence:**
Wenneng Wu
wuwenneng123@126.com
Pei Li
pl19890627@126.com
Heng Luo
luoheng@gzcnp.cn

Supplementary data

1. ^{1}H NMR, ^{13}C NMR and HRMS spectral data for synthesized compounds

2-Chloro-*N*-(3-((6-(trifluoromethyl)pyrimidin-4-yl)oxy)phenyl)benzamide (**5b**). White solid; yield 57.9%; m.p. 106-108 °C; ^{1}H NMR (600 MHz, DMSO-d$_6$, ppm) *δ:* 10.75 (s, 1H,-CONH-), 9.00 (s, 1H, pyrimidine-H), 7.79 (t, 2H, *J* = 1.8 Hz, Ph-H), 7.62-57 (m, 3H, Ph-H), 7.54-7.51 (m, 1H, Ph-H), 7.49 (t, 2H, *J* = 7.8 Hz, Ph-H), 7.06 (dd, 1H, J_1 = 1.8 Hz, J_2 = 8.4 Hz, Ph-H); ^{13}C NMR (125 MHz, DMSO-d$_6$, ppm) *δ*: 170.57, 165.60, 159.81, 153.15 (q, *J* = 35.4 Hz), 152.30, 140.87, 137.15, 131.71, 130.58, 130.39, 130.16, 129.42, 127.75, 121.81 (q, *J* = 272.8 Hz), 117.62, 117.44, 113.05, 106.75; HRMS (ESI) caculated for $C_{18}H_{11}O_2N_3ClF_3$ $[M+Na]^+$: 416.03818, found: 416.03823.

3-Methoxy-*N*-(3-((6-(trifluoromethyl)pyrimidin-4-yl)oxy)phenyl)benzamide (**5c**). White solid; yield 30.8%; m.p. 119-121 °C; ^{1}H NMR (600 MHz, DMSO-d$_6$, ppm) *δ*: 10.42 (s, 1H, -CONH-), 8.99 (s, 1H, pyrimidine-H), 7.83 (d, 1H, *J* = 2.4 Hz, Ph-H), 7.77 (s, 1H, pyrimidine-H), 7.71 (d, 1H, *J* = 7.8 Hz, Ph-H), 7.55 (d, 1H, *J* =7.8 Hz, Ph-H), 7.50-7.44 (m, 2H, Ph-H), 7.50 (t, 1H, *J* = 7.8 Hz, Ph- H), 7.18 (dd, 1H, J_1 = 1.8 Hz, J_2 = 9.0 Hz, Ph-H), 7.04 (dd, 1H, J_1 = 3.0 Hz, J_2 = 7.2 Hz, Ph-H), 3.84 (s, 3H, CH_3O-); ^{13}C NMR (125 MHz, DMSO-d$_6$, ppm) *δ*: 170.61, 165.95, 159.84, 159.66, 156.14 (q, *J* = 34.8 Hz), 152.21, 141.13, 136.54, 130.36, 130.05, 121.81 (q, *J* = 273.15

Hz), 120.35, 118.36, 117.88, 117.17, 113.78, 113.46, 106.66, 60.58, 55.79; HRMS (ESI) caculated for $C_{19}H_{14}O_3N_3F_3$ $[M+Na]^+$: 412.08731, found: 412.08795.

4-(Tert-butyl)-*N*-(3-((6-(trifluoromethyl)pyrimidin-4-yl)oxy)phenyl)benzamide (**5d**). White solid; yield 68.2%; m.p. 120-122 °C; ^{1}H NMR (DMSO-d_6, 600 MHz, ppm) *δ*: 9.79 (s, 1H, -CONH-), 8.64 (s, 1H, pyrimidine-H), 7.92 (d, 2H, *J* = 8.4 Hz, Ph-H), 7.83 (d, 1H, *J* = 7.2 Hz, Ph-H), 7.56 (d, 2H, *J* = 9.0 Hz, Ph-H), 7.38-7.32 (m, 3H, Ph-H), 7.08 (s, 1H, pyrimidine-H), 1.31 (s, 9H); ^{13}C NMR (DMSO-d_6, 150 MHz, ppm) δ: 164.18, 162.12, 159.43, 152.91 (q, *J* = 34.2 Hz), 144.53, 132.39, 130.67, 126.70, 126.27, 124.12, 122.21 (q, *J* = 272.55 Hz), 121.83, 114.54, 56.06; HRMS (ESI) caculated for $C_{22}H_{21}O_2N_3F_3$ $[M+Na]^+$: 438.13953, found: 438.13998.

3-(Trifluoromethyl)-*N*-(3-((6-(trifluoromethyl)pyrimidin-4-yl)oxy)phenyl)benzamide (**5e**). White solid; yield 39.8%; m.p. 115-117 °C; ^{1}H NMR (600 MHz, DMSO-d_6, ppm) *δ*: 10.66 (s, 1H， -CONH-), 8.99 (s, 1H, Pyrimidine-H), 8.30 (s, 1H, Ph-H), 7.79 (s, 1H, Pyrimidine-H), 8.27 (d, 2H, *J* = 7.8 Hz, Ph-H), 7.99 (d, 1H, J = 7.8 Hz, Ph-H), 7.81-7.80 (m, 2H, Ph-H), 7.71 (d, 1H, *J* = 7.8 Hz, Ph-H), 7.62 (t, 1H, J = 7.8 Hz, Ph-H), 7.51 (t, 1H, *J* = 7.8 Hz, Ph-H), 7.08 (dd, 1H, J_1 = 1.8 Hz, J_2 = 7.8 Hz, Ph-H); ^{13}C NMR (150 MHz, DMSO-d_6, ppm) δ: 170.60, 164.72, 159.83, 156.13 (q, *J* = 35.1 Hz), 152.22, 140.80, 136.00, 132.35, 130.47, 130.24, 129.79 (q, *J* = 32.1 Hz), 128.78, 125.32 (q, *J* = 271.2 Hz), 124.73, 121.81 (q, *J* = 272.8 Hz), 118.50, 117.55, 113.97, 106.74; HRMS (ESI) caculated for $C_{19}H_{11}O_{22}N_3F_6$ $[M+H]^+$: 450.06418, found: 450.06472.

4-Methoxy-3-(trifluoromethyl)-*N*-(3-((6-(trifluoromethyl)pyrimidin-4-yl)oxy)phenyl)benzamide (**5f**). White solid; yield 40.4%; m.p. 108-110 °C; 1H NMR (600 MHz, DMSO-d_6, ppm) δ: 10.49 (s, 1H, -CONH-), 8.99 (s, 1H, Pyrimidine-H), 8.29 (dd, 1H, J_1 = 1.8 Hz, J_2 = 9.0 Hz, Ph-H), 8.25 (s, 1H, Ph-H), 7.80 (t, 1H, *J* = 1.8 Hz, Ph-H), 7.76 (s, 1H, Pyrimidine-H), 7.70 (d, 1H, *J* = 8.4 Hz, Ph-H), 7.49 (t, 1H, *J* = 7.8 Hz, Ph-H), 7.43 (d, 1H, *J* = 9.0 Hz, Ph-H), 7.04 (dd, 1H, J_1 = 2.4 Hz, J_2 = 8.4 Hz, Ph-H), 3.99 (s, 3H); ^{13}C NMR (150 MHz, DMSO-d_6, ppm) *δ*: 170.60, 164.72, 159.83, 156.13 (q, J = 34.8 Hz), 152.21, 141.04, 134.59, 130.36, 127.03, 126.69, 124.79 (q, J = 270.6 Hz), 121.18 (q, J = 273.3 Hz), 118.42, 117.21, 113.88, 113.24, 106.67, 57.07; HRMS (ESI) caculated for $C_{18}H_{10}O_2N_3BrF_4$ $[M+Na]^+$: 480.07501, found: 480.07533.

3-Bromo-4-chloro-*N*-(3-((6-(trifluoromethyl)pyrimidin-4-yl)oxy)phenyl)benzamide (**5g**). White solid; yield 52.4%; m.p. 146-148 °C; ^{1}H NMR (600 MHz, DMSO-d_6, ppm) *δ*: 10.56 (s, 1H, -CONH-), 8.98 (s, 1H, 8.99 (s, 1H, Pyrimidine-H)), 8.34 (d, 1H, *J* = 1.8 Hz, Ph-H), 7.97 (dd, 1H, J_1 = 1.8 Hz, J_2 = 7.8 Hz), 7.81 (d, 1H, *J* = 8.4 Hz), 7.78 (s, 2H, Pyrimidine-H and Ph-H), 7.67 (d, 1H, *J* = 8.4 Hz, Ph-H), 7.49 (t, 1H, *J* = 8.4 Hz, Ph-H), 7.06 (dd, 1H, J_1 = 1.8 Hz, J_2 = 7.8 Hz, Ph-H); ^{13}C NMR (125 MHz, DMSO-d_6, ppm) δ: 170.09, 163.24, 159.33, 155.63 (q, *J* = 35.6 Hz), 151.71, 140.24, 136.56, 134.86, 132.74, 130.58, 129.97, 128.64, 121.61, 121.37 (q, *J* = 273.2 Hz), 117.93, 113.39, 106.23; HRMS (ESI) caculated for $C_{18}H_{10}O_2N_3BrClF_4$ $[M+Na]^+$: 193.94843, found: 493.94892.

5-Bromo-2-fluoro-*N*-(3-((6-(trifluoromethyl)pyrimidin-4-yl)oxy)phenyl)benzamide (**5h**). White solid; yield 42.5%; m.p. 106-108 °C; ^{1}H NMR (600 MHz, DMSO-d_6, ppm) δ: 10.73 (s, 1H, -CONH-), 8.98 (s, 1H, Pyrimidine-H), 7.06 (dd, 1H, J_1 = 2.4 Hz, J_2 = 6.0 Hz), 7.80-7.77 (m, 2H, Pyrimidine-H and Ph-H), 7.75 (t, 1H, *J* = 1.8 Hz, Ph-H),

7.59 (d, 1H, J = 8.4 Hz, Ph-H), 7.50 (t, 1H, J = 8.4 Hz, Ph-H), 7.39 (t, 1H, J = 9.0 Hz, Ph-H); ^{13}C NMR (125 MHz, DMSO-d_6, ppm) δ: 170.56, 161.91, 159.43, 157.77, 156.11 (q, J = 35.0 Hz), 152.27, 140.55, 135.71, 132.62, 130.62, 127.26, 121.81 (q, J = 273.4 Hz), 119.24, 119.08, 117.85, 117.68, 116.61, 113.32, 106.77; HRMS (ESI) caculated for $C_{18}H_{10}O_2N_3BrF_4$ $[M+Na]^+$: 477.97797, found: 477.97847.

3,4,5-Trimethoxy-*N*-(3-((6-(trifluoromethyl)pyrimidin-4-yl)oxy)phenyl)benzamide (**5i**). White solid; yield 56.4%; m.p. 125-127 °C; ^{1}H NMR (600 MHz, DMSO-d_6, ppm) δ: 10.31 (s, 1H, -CONH-), 8.99 (s, 1H, Pyrimidine-H), 8.34 (d, 1H, J = 1.8 Hz, Ph-H), 7.97 (dd, 1H, J_1 = 1.8 Hz, J_2 = 7.8 Hz), 7.81 (d, 1H, J = 8.4 Hz), 7.78 (s, 1H, Pyrimidine-H), 7.76 (t, 1H, J = 1.8 Hz, Ph-H), 7.69 (d, 1H, J = 8.4 Hz, Ph-H), 7.50 (t, 1H, J = 7.8 Hz, Ph-H), 7.28 (s, 2H, Ph-H), 7.05 (dd, 1H, J_1 = 1.8 Hz, J_2 = 7.8 Hz), 3.88 (s, 6H), 3.74 (s, 3H); ^{13}C NMR (150 MHz, DMSO-d_6, ppm) δ: 170.62, 165.58, 159.85, 155.63 (q, J = 35.2 Hz), 153.11, 152.22, 141.08, 140.93, 130.37, 130.22, 121.37 (q, J = 273.4 Hz), 118.54, 117.19, 113.97, 106.69, 105.82, 60.58, 56.56; HRMS (ESI) caculated for $C_{21}H_{18}O_5N_3F_3$ $[M+Na]^+$: 472.10855, found: 472.10901.

***N*-(4-((6-(trifluoromethyl)pyrimidin-4-yl)oxy)phenyl)benzamide** (**5j**). White solid; yield 48.6%; m.p. 205-207 °C; ^{1}H NMR (600 MHz, DMSO-d_6, ppm) δ: 10.40 (s, 1H,-CONH-), 8.98 (s, 1H, Pyrimidine-H), 7.98 (d, 2H, J = 7.2 Hz, Ph-H), 7.89 (d, 2H, J = 8.4 Hz), 7.75 (s, 1H, Pyrimidine-H), 7.62 (t, 1H, J = 7.2 Hz, Ph-H), 7.57 (t, 2H, J = 8.4 Hz, Ph-H), 7.29 (d, 2H, J = 8.4 Hz, Ph-H); ^{13}C NMR (150 MHz, DMSO-d_6, ppm) δ: 170.84, 166.06, 159.83, 156.02 (q, J = 35.1 Hz), 147.77, 137.65, 135.28, 132.13, 128.89, 128.15, 122.23, 122.13, 121.84 (q, J = 272.8 Hz), 106.57; HRMS (ESI) caculated for $C_{18}H_{12}O_2N_3F_3$ $[M+Na]^+$: 382.07681, found: 382.07738.

3-Methyl-*N*-(4-((6-(trifluoromethyl)pyrimidin-4-yl)oxy)phenyl)benzamide (**5k**). White solid; yield 50.2%; m.p. 179-181 °C; ^{1}H NMR (600 MHz, DMSO-d_6, ppm) δ: 10.31 (s, 1H, -CONH-), 8.98 (s, 1H, Pyrimidine-H), 7.78 (d, 2H, J = 9.0 Hz, Ph-H), 7.79 (s, 1H, Pyrimidine-H), 7.66 (d, 2H, J = 7.2 Hz), 7.74 (s, 1H, Ph-H), 7.45-7.41 (m, 2H, Ph-H), 7.28 (d, 2H, J = 9.0 Hz, Ph-H), 2.42 (s, 3H, CH_3-); ^{13}C NMR (150 MHz, DMSO-d_6, ppm) δ: 170.84, 166.17, 159.82, 156.03 (q, J = 35.4 Hz), 147.72, 138.21, 137.69, 135.30, 132.69, 128.78, 128.61, 125.30, 122.21, 122.10, 121.83 (q, J = 273.0 Hz), 106.56, 21.43; HRMS(ESI) caculated for $C_{19}H_{15}F_3N_3O$ $[M+H]^+$: 374.11132; found: 374.11141

3-Fluoro-*N*-(4-((6-(trifluoromethyl)pyrimidin-4-yl)oxy)phenyl)benzamide (**5l**). White solid; yield 45.2%; m.p. 192-194 °C; ^{1}H NMR (600 MHz, DMSO-d_6, ppm) δ: 10.45 (s, 1H, -CONH-), 8.97(s, 1H, Pyrimidine-H), 7.88(d, 2H, J = 9.0 Hz, Ph-H), 7.84 (d, 1H, J=7.8 Hz, Ph-H), 7.79 (d, 2H, J = 7.8 Hz, Ph-H), 7.74 (s, 1H, Pyrimidine-H), 7.62 (q, 1H, J = 7.8 Hz, Ph-H), 7.47 (t, 1H, J = 8.4 Hz, Ph-H); ^{13}C NMR (150 MHz, DMSO-d_6, ppm) δ: 170.80, 164.64, 163.22(d, J = 242.85 Hz), 159.81, 156.04 (q, J = 35.3 Hz), 147.96, 137.58(d, J = 7.08 Hz), 137.35, 131.11(d, J = 7.8 Hz), 124.38, 122.28(d, J = 5.55 Hz), 121.82(q, J = 273.15 Hz), 121.28, 120.00, 119.09(d, J = 21.15 Hz), 115.05(d, J = 22.65 Hz), 106.56; HRMS (ESI) caculated for $C_{18}H_{11}O_2N_3F_4$ $[M+Na]^+$: 400.06738, found: 400.06796.

2-Chloro-*N*-(4-((6-(trifluoromethyl)pyrimidin-4-yl)oxy)phenyl)benzamide (**5m**). White solid; yield 29.5%; m.p. 226-228 °C; ^{1}H NMR (600 MHz, DMSO-d_6, ppm) δ:

10.65 (s, 1H, -CONH-), 8.97 (s, 1H, Pyrimidine-H), 7.88 (d, 2H, J = 9.0 Hz, Ph-H), 7.75 (s, 1H, Pyrimidine-H), 7.62 (q, 2H, *J* = 7.8 Hz, Ph-H), 7.53 (t, 1H, *J* = 7.8 Hz, Ph-H), 7.62 (t, 1H, *J* = 6.6 Hz, Ph-H), 7.48 (t, 1H, *J* = 7.2 Hz, Ph-H), 7.29 (d, 1H, *J* = 9.0 Hz, Ph-H); ^{13}C NMR (150 MHz, DMSO-d$_6$, ppm) δ: 170.84, 165.41, 159.80, 156.03 (q, *J* = 35.4 Hz), 147.86, 137.32, 131.65, 129.41, 127.77, 122.46, 121.83 (q, *J* = 273.0 Hz), 121.36, 106.59; HRMS (ESI): caculated for $C_{18}H_{11}O_2N_3ClF_3$ [M+Na]$^+$: 416.03818, found: 416.03841.

4-Chloro-*N*-(4-((6-(trifluoromethyl)pyrimidin-4-yl)oxy)phenyl)benzamide (**5n**). White solid; yield 30.2%; m.p. 254-255 °C; ^{1}H NMR (600 MHz, DMSO-d$_6$, ppm) *δ*: 10.45 (s, 1H, -CONH-), 8.97 (s, 1H, Pyrimidine-H), 7.78 (d, 2H, *J* = 7.2 Hz, Ph-H), 7.79 (s, 1H, Pyrimidine-H), 7.63 (d, 2H, *J* = 7.8 Hz, Ph-H), 7.29 (d, 2H, *J* = 7.2 Hz), 2.42 (s, 3H); ^{13}C NMR (150 MHz, DMSO-d$_6$, ppm) δ: 170.81, 164.93, 159.81, 156.02 (q, *J* = 35.1 Hz), 147.89, 137.43, 136.97, 133.94, 130.11, 128.97, 122.27, 122.21, 121.82 (q, J = 273.3 Hz), 106.58; HRMS (ESI) caculated for $C_{18}H_{11}O_2N_3ClF_3$ [M+Na]$^+$: 416.03818, found: 416.03834.

4-Methoxy-*N*-(4-((6-(trifluoromethyl)pyrimidin-4-yl)oxy)phenyl)benzamide (**5o**). White solid; yield 41.6%; m.p. 175-176 °C; ^{1}H NMR (600 MHz, DMSO-d$_6$, ppm) *δ*: 10.23 (s, 1H,-CONH-), 8.98 (s, 1H, Pyrimidine-H), 7.99 (d, 2H, *J* = 8.4 Hz, Ph-H), 7.87 (d, 2H, *J* = 9.0 Hz, Ph-H), 7.75 (s, 1H, Pyrimidine-H), 7.27 (d, 2H, *J* = 9.0 Hz, Ph-H), 7.09 (d, 2H, *J* = 8.4 Hz, Ph-H), 3.85 (s, 3H,CH_3O-); ^{13}C NMR (150 MHz, DMSO-d$_6$, ppm) *δ*: 170.85, 164.40, 159.83, 156.00 (q, *J* = 35.4 Hz), 147.59, 137.82, 130.09, 127.25, 122.17, 122.07, 121.84 (q, *J* = 273.3 Hz), 114.10, 106.57, 55.59; HRMS (ESI) caculated for $C_{19}H_{14}O_3N_3F_3$ [M+Na]$^+$: 412.08731, found: 412.08787.

4-(Tert-butyl)-*N*-(4-((6-(trifluoromethyl)pyrimidin-4-yl)oxy)phenyl)benzamide (**5p**). White solid; yield 37.7%; m.p. 167-168 °C; ^{1}H NMR (600 MHz, DMSO-d$_6$, ppm) *δ*: 10.32 (s, 1H, −CONH−), 8.97 (s, 1H, Pyrimidine-H), 7.92 (q, 4H, *J* = 8.4 Hz, Ph-H), 7.72 (s, 1H, Pyrimidine-H), 7.56 (q, 2H, *J* = 7.8 Hz, Ph-H), 7.27 (d, 2H, *J* = 9.0 Hz, Ph-H), 1,32 (s, 9H); ^{13}C NMR (150 MHz, DMSO-d$_6$, ppm) *δ*: 170.84, 166.01,159.82, 156.04 (q, *J* = 34.95 Hz), 154.97, 147.66, 137.76, 132.59, 122.18, 128.02, 121.82 (q, *J* = 273.15 Hz), 125.63, 122.18, 106.48, 35.12, 31.36; HRMS (ESI) caculated for $C_{22}H_{21}O_2N_3F_3$ [M+Na]$^+$: 438.13953, found: 438.13985.

4-Amino-*N*-(4-((6-(trifluoromethyl)pyrimidin-4-yl)oxy)phenyl)benzamide (**5q**). White solid; yield 22.6%; m.p. 205-207 °C; ^{1}H NMR (600 MHz, DMSO-d$_6$, ppm) *δ*: 9.89 (s, 1H, -CONH-), 8.97 (s, 1H, Pyrimidine-H), 7.85 (d, 2H, *J* = 9.0 Hz, Ph-H), 7.74 (s, 1H, Pyrimidine-H), 7.73 (d, 2H, *J* = 8.4 Hz, Ph-H), 7.23 (d, 2H, *J* = 8.4 Hz, Ph-H), 6.62 (d, 2H , *J* = 8.4 Hz, Ph-H), 5.78 (s, 2H, -NH_2); ^{13}C NMR (150 MHz, DMSO-d$_6$, ppm) *δ*: 171.02, 165.91, 155.99 (q, *J* = 35.25 Hz), 152.71, 147.34, 137.99, 129.86, 122.03, 121.88 (q, *J* = 273.6 Hz), 121.81, 121.33, 113.02, 106.49; HRMS (ESI) caculated for $C_{18}H_{15}O_2N_4F_3$ [M+Na]$^+$: 397.08777, found: 382.08828.

3-(Trifluoromethyl)-*N*-(4-((6-(trifluoromethyl)pyrimidin-4-yl)oxy)phenyl)benzamide (**5r**). White solid; yield 25.6%; m.p. 175-177 °C; ^{1}H NMR (600 MHz, DMSO-d$_6$, ppm) *δ*: 10.62 (s, 1H, -CONH-), 8.98 (s, 1H, Pyrimidine-H), 8.18 (d, 2H, *J* = 7.8 Hz, Ph-H), 7.94 (d, 2H, *J* = 7.8 Hz, Ph-H), 7.90 (d, 2H, *J* = 9.0 Hz, Ph-H), 7.75 (s, 1H, Pyrimidine-H), 7.31 (d, 2H, *J* = 9.0 Hz, Ph-H); ^{13}C NMR (150 MHz, DMSO-d$_6$, ppm)

δ: 170.79, 164.88, 159.81, 156.05 (q, *J* = 35.4 Hz), 148.03, 139.06, 137.29, 132.03 (q, *J* = 30.6 Hz), 129.08, 125.88, 122.35 (q, *J* = 270.4 Hz), 122.32, 122.25, 121.81 (q, *J* = 273.4 Hz), 106.57; HRMS (ESI) caculated for $C_{19}H_{11}O_2N_3F_6$ $[M+H]^+$: 450.06418, found: 450.06468.

4-(Trifluoromethyl)-*N*-(4-((6-(trifluoromethyl)pyrimidin-4-yl)oxy)phenyl)benzamide (**5s**). White solid; yield 25.6%; m.p. 200-202 °C; ^{1}H NMR (600 MHz, DMSO-d_6, ppm) δ: 10.61 (s, 1H, -CONH-), 8.98 (s, 1H, Pyrimidine-H), 8.18 (d, 2H, *J* = 7.8 Hz, Ph-H), 7.95 (d, 2H, *J* = 7.8 Hz, Ph-H), 7.89 (d, 2H, *J* = 8.4 Hz, Ph-H), 7.76 (s, 1H, Pyrimidine-H), 7.31 (d, 2H, *J* = 8.4 Hz, Ph-H); ^{13}C NMR (150 MHz, DMSO-d_6, ppm) *δ*: 170.72, 164.81, 159.82, 156.02 (q, *J* = 34.5 Hz), 151.46, 148.04, 139.08, 137.14, 132.01 (q, *J* = 31.5 Hz), 129.10, 125.93, 125.29 (q, *J* = 270.4 Hz), 122.35, 122.25, 121.83 (q, *J* = 273.4 Hz), 106.63; HRMS (ESI) caculated for $C_{19}H_{11}O_{22}N_3F_6$ $[M+H]^+$: 450.06418, found: 450.06477.

2,6-Dimethoxy-*N*-(4-((6-(trifluoromethyl)pyrimidin-4-yl)oxy)phenyl)benzamide (**5t**). White solid; yield 39.2%; m.p. 127-128 °C; ^{1}H NMR (600 MHz, DMSO-d_6, ppm) δ: 10.36 (s, 1H, -CONH-), 8.96 (s, 1H, Pyrimidine-H), 7.82 (d, 2H, *J* = 7.2 Hz, Ph-H), 7.72 (s, 1H, Pyrimidine-H), 7.38 (t, 1H, *J* = 8.4 Hz, Ph-H), 7.23 (d, 2H, *J* = 9.0 Hz, Ph-H), 6.73 (d, 2H, *J* = 8.4 Hz, Ph-H), 3.78 (s, 6H, CH_3O-); ^{13}C NMR (150 MHz, DMSO-d_6, ppm) *δ*: 171.02, 159.64, 156.02 (q, *J* = 34.95 Hz), 147.33, 137.97, 130.86, 122.27, 121.83(q, *J* = 273.3 Hz), 120.62, 116.98, 106.47, 104.45, 56.26; HRMS (ESI) caculated for $C_{20}H_{16}O_4N_3F_3$ $[M+H]^+$: 442.09790, found: 442.09851.

3,4-Dimethoxy-*N*-(4-((6-(trifluoromethyl)pyrimidin-4-yl)oxy)phenyl)benzamide (**5u**). White solid; yield 39.2%; m.p. 168-170 °C; ^{1}H NMR (600 MHz, DMSO-d_6, ppm) *δ*: 10.21 (s, 1H, -CONH-), 8.98 (s, 1H, Pyrimidine-H), 7.78 (d, 2H, *J* = 8.4 Hz, Ph-H), 7.73 (s, 1H, Pyrimidine-H), 7.66 (d, 1H, *J* = 7.8 Hz, Ph-H), 7.56 (s, 1H, Ph-H), 7.28 (d, 2H, *J* = 8.4 Hz, Ph-H), 7.11 (d, 1H, *J* = 8.4 Hz, Ph-H), 3.86 (s, 3H, CH_3O-), 3.85 (s, 3H); ^{13}C NMR (150 MHz, DMSO-d_6, ppm) *δ*: 170.84, 165.43, 159.83, 156.26 (q, *J* = 34.8 Hz), 152.19, 148.81, 147.65, 137.77, 127.29, 122.21, 122.16, 121.83 (q, *J* = 272.8 Hz), 121.52, 111.54, 111.36, 106.54, 56.14, 56.10; HRMS (ESI) caculated for $C_{20}H_{16}O_4N_3F_3$ $[M+H]^+$: 442.09787, found: 442.09851.

3-Bromo-5-(trifluoromethyl)-*N*-(4-((6-(trifluoromethyl)pyrimidin-4-yl)oxy)phenyl)benzamide (**5v**). White solid; yield 20.4%; m.p. 132-133 °C; ^{1}H NMR (600 MHz, DMSO-d_6, ppm) *δ*: 10.66 (s, 1H, -CONH-), 8.97 (s, 1H, Pyrimidine-H), 8.47 (s, 1H, Ph-H), 8.30 (s, 1H, Ph-H), 8.24 (s, 1H, Ph-H), 7.86 (d, 2H, *J* = 9.0 Hz, Ph-H), 7.75 (s, 1H, Pyrimidine-H), 7.30 (d, 2H, *J* = 9.0 Hz, Ph-H); ^{13}C NMR (150 MHz, DMSO-d_6, ppm) *δ*: 170.79, 163.03, 159.81, 156.04 (q, *J* = 32.25 Hz), 148.19, 138.07, 136.99, 134.99, 131.6 (q, *J* = 32.7 Hz), 131.38, 123.02, 124.34 (q, *J* = 271.95 Hz), 124.10, 121.82 (q, *J* = 272.85 Hz), 106.61; HRMS (ESI) caculated for $C_{19}H_9O_2N_3BrF_6$ $[M+Na]^+$: 527.97412, found: 529.97205.

3,4,5-Trimethoxy-*N*-(4-((6-(trifluoromethyl)pyrimidin-4-yl)oxy)phenyl)benzamide (**5w**). White solid; yield 27.5%; m.p. 169-171 °C; ^{1}H NMR (600 MHz, DMSO-d_6, ppm) *δ*: 10.25 (s, 1H, -CONH-), 8.98 (s, 1H, Pyrimidine-H), 7.84 (d, 2H, *J* = 9.0 Hz, Ph-H), 7.75 (s, 1H, Pyrimidine-H), 7.31 (t, 2H, *J* = 8.4 Hz, Ph-H), 7.30 (s, 2H, Ph-H), 3.88 (s, 6H, CH_3O-), 3.74 (s, 3H, CH_3O-); ^{13}C NMR (150 MHz, DMSO-d_6, ppm) *δ*: 170.83,

165.42, 156.03 (q, J = 35.1 Hz), 153.12, 147.84, 137.51, 130.37, 122.38, 122.23, 121.83 (q, J = 273.0 Hz), 106.59, 105.80, 60.60, 56.59; HRMS (ESI) caculated for $C_{21}H_{18}O_5N_3F_3$ $[M+Na]^+$: 472.10855, found: 472.10908.

2. The crystallographic parameters of compounds 5q

Table S1. Crystal data of partly of the target compound **5q**

Compound		**5q**	
Empirical formula	$C_{22}H_{22}F_3N_3O_3$	μ/mm^{-1}	0.106
Formula weight	433.43	F(000)	904.00
Temperature/K	298(2)	Crystal size/mm^3	0.44× 0.38 × 0.30
Crystal system	Monoclinic	Radiation	MoKα (λ = 0.71073)
Space group	P2(1)/c	2Θ range for data collection/°	4.144 to 45.458
a/Å	17.2341(15)	Index ranges	$-20 \le h \le 20$, $-12 \le k \le 14$, $-12 \le l \le 10$
b/Å	12.1803(11)	Reflections collected	10727
c/Å	10.5015(9)	Independent reflections	1985 [R_{int} = 0.0432, R_{sigma} = 0.0510]
α/°	90.0	Data/restraints/parameters	3855/0/342
β/°	97.021(2)	Goodness-of-fit on F^2	1.000
γ/°	90.00	Final R indexes [I>=2σ (I)]	R_1 = 0.1469, wR_2 = 0.2676
Volume/$Å^3$	2187.9(3)	Final R indexes [all data]	R_1 = 0.0771, wR_2 = 0.2218
Z	4	Largest diff. peak/hole / e $Å^{-3}$	0.52/-0.31
$\rho_{calc}g/cm^3$	1.316		
